# Health care providers’ perspectives on providing end-of-life psychiatric care in cardiology and oncology hospitals: a cross-sectional questionnaire survey

**DOI:** 10.1186/s12904-023-01138-z

**Published:** 2023-03-15

**Authors:** Kanako Ichikura, Shiho Matsuoka, Hiroki Chiba, Hina Ishida, Yuko Fukase, Hanako Murase, Hirokuni Tagaya, Takashi Takeuchi, Eisuke Matsushima

**Affiliations:** 1 Department of Health Science, Kitasato University School of Allied Health Sciences, Kanagawa, Japan; 2 Department of Clinical Neuropsychology, Kitasato University Graduate School of Medical Sciences, Kanagawa, Japan; 3 Section of Liaison Psychiatry and Palliative Medicine, Graduate School of Medical and Dental Sciences, Tokyo Medical and Dental University, Tokyo, Japan; 4 Department of Medical Education, Kitasato University School of Medicine, Kanagawa, Japan; 5 1-15-1 Kitasato, Minami-ku, 252-0373 Sagamihara, Kanagawa Japan

**Keywords:** End-of-life, Terminal, Palliative care, Psychiatric care, Psychological care, Distress

## Abstract

**Background:**

Psychological distress is a major concern for patients with end-stage heart failure (HF). However, psychiatric care for patients with HF is not as organized as that for patients with cancer. Therefore, the aim of this study was to elucidate and compare the barriers faced by health care providers of cardiology and oncology hospitals in providing end-of-life psychiatric care to patients with HF and cancer, respectively.

**Methods:**

We conducted a cross-sectional questionnaire survey among the health care providers of Japan. Questionnaires were mailed to physicians and nurses of 427 cardiology and 347 oncology hospitals in March 2018 to assess health care providers’ perspectives. First, we compared the scores of the Palliative Care Difficulties Scale and the original scale of end-of-life psychiatric care difficulties between health care providers of cardiology and oncology hospitals. Second, we asked the health care providers to describe the barriers to providing end-of-life psychiatric care with an open-ended question and then compared the freely-provided descriptions using content analysis.

**Results:**

A total of 213 cardiology and 224 oncology health care providers responded to the questionnaire. No significant differences were found between health care providers of cardiology and oncology hospitals in the frequency of experiencing barriers to providing end-of-life psychiatric care (59.8% and 62.2%, respectively). A content analysis identified the following eight barriers: “patients’ personal problems,” “family members’ problems,” “professionals’ personal problems,” “communication problems between professionals and patients,” “problems specific to end-of-life care,” “problems specific to psychiatric care,” “problems of institution or system,” and “problems specific to non-cancer patients.” The “problems specific to noncancer patients” was described more frequently by health care providers in cardiology hospitals than that in oncology hospitals. However, there were no significant differences in other items between the two.

**Conclusion:**

Although health care providers of both cardiology and oncology hospitals faced barriers to providing end-of-life psychiatric care, those of cardiology hospitals particularly faced challenges pertaining to non-cancer patients, such as unpredictability of prognosis or insufficiency of guideline development. A system of psychiatric care, specifically for patients with HF, should be established.

**Supplementary Information:**

The online version contains supplementary material available at 10.1186/s12904-023-01138-z.

## Background

Heart failure (HF) is potentially fatal, unless a heart transplantation is performed, and it is a serious healthcare and economic burden on patients and their caregivers. The World Health Organization estimated the worldwide mortality from cardiovascular disease at 15.2 million in 2016 [1], making it the most common cause of death (40%) among middle-aged and older adults [2]. Despite the recent rapid progress in medical treatments, the median survival rate after patients’ first hospitalization is low in severe HF (2.1 years) [3]. In addition, HF has inflicted a burden of $180 million on the global health system [4].

Patients with advanced HF commonly experience psychological symptoms, the most common of which are depression and anxiety, as well as physical symptoms, such as dyspnea, pain, or fatigue [5, 6]. Severe clinical depression is diagnosed in 12 to 33% of all patients with heart disease [7, 8] and in 38 to 42% of those with severe HF, featuring New York Heart Association class III-IV symptoms [9]. Among patients with HF, 29% exhibit severe and clinically significant anxiety symptoms, and 9% have anxiety disorders, including generalized anxiety disorders [6, 10]. In addition, psychological symptoms have a highly negative impact on the quality of life and are associated with poor treatment adherence, severe physical symptoms, long-term hospitalization, and a reduced survival rate [11]. Therefore, psychological symptoms, such as depression or anxiety, are particularly challenging problems for patients with end-stage HF [12, 13].

Psychiatric care, including pharmacotherapy and psychotherapy, can be of benefit for patients with HF who have psychological symptoms. However, there is inadequate evidence for the efficacy of pharmacotherapy in patients with HF [6, 14], and psychiatric pharmacotherapy, such as antidepressants, increases the risk of all-cause death among HF patients [15]. Nevertheless, psychotherapy has received attention among patients with HF in recent years, and cognitive behavioral therapy in particular has been shown to improve psychological symptoms [16, 17]. Relaxation, meditation, and mindfulness-based psychoeducation can also alleviate these symptoms [18, 19]. However, there is limited evidence and guidance on the efficacy of such psychiatric care among patients with terminal HF [20, 21].

In patients with end-stage cancer, many of whom experience psychological symptoms similar to patients with end-stage HF, many studies have demonstrated the effectiveness of pharmacotherapy and psychotherapy [22–24]. Workshops or guidelines for oncologists can also enhance their practical skills in providing end-of-life psychiatric care [25, 26]. A comparison between the difficulties in providing psychiatric care for patients with end-stage HF versus those with cancer could provide useful insights into potential barriers to providing psychiatric care for patients with end-stage HF. However, to date, no study has examined the barriers to providing psychiatric care for patients with HF. In addition, we believe that a qualitative study design, examining thee difficulties faced by health care providers in pain management, would be also helpful in investigating the difficulties with psychiatric management and identifying the barriers to providing psychiatric care [27].

The aims of this study were to identify and compare the barriers faced by health care providers of cardiology and oncology hospitals in providing psychiatric care to end-of-life patients.

## Methods

### Design and participants

This was a national, cross-sectional survey conducted among Japanese health care providers of cardiology and oncology hospitals using self-completed questionnaires. We mailed the questionnaires to the departments of cardiovascular internal medicine of 427 implantable cardioverter defibrillators (ICD) specialized hospitals and to the departments of respiratory medicine of 347 designated cancer hospitals; we asked them to deliver the questionnaires directly to the chief physicians and the chief nurses in each department in March 2018. ICD specialized hospitals are equipped to perform implantation of ICDs and are the center of cardiovascular medicine in Japan. Additionally, designated cancer hospitals, recommended by the prefectural governments, can provide high-quality cancer treatment, as guaranteed by the Ministry of Health, Labour and Welfare in Japan. These medical facilities provide palliative care by a team of medical professional, provide specialized cancer treatments, establish local cooperation systems for cancer treatments, and provide consultation, support, and information for cancer patients.

### Demographic and clinical characteristics

We collected demographic and clinical information from the self-completed questionnaires. First, we included the following data: sex, age, and medical license of the staff of each health care provider. Second, we included the following data: area (Hokkaido/Tohoku, Kanto/Koshinetsu, Chubu/Hokuriku, Kinki, Chugoku/Shikoku, and Kyushu/Okinawa area), hospital type (national medical center, academic medical center, general hospital except academic medical center, specialized hospital), the number of hospital beds, and the presence of a palliative care unit, palliative care team, liaison psychiatry team, palliative care physicians, psychiatrists, and psychologists at hospitals.

### Outcome measures

#### Difficulty in providing palliative care

The Palliative Care Difficulties Scale—a 15-item self-reported scale—was developed in Japan [28]. The responses are scored in the format of a 4-point Likert-type scale ranging from 0 to 3 (overall score range: 0–42). The scale contains of the following five factors, each having three items: (1) alleviating symptoms, (2) expert support, (3) multidisciplinary communication, (4) communication with patient/family, and (5) community coordination. The reliability and validity of this measure were sufficiently supported in an earlier study [29].

#### Difficulty in providing end-of-life psychiatric care

We developed the following original question (Sup.1) for assessing the difficulty in providing end-of-life psychiatric care: “Do you face challenges in providing psychiatric care for patients at their end of life?” The possible answers were “yes” or “no.”

#### Barriers to providing end-of-life psychiatric care

To identify the barriers to providing end-of-life psychological care, we asked the following original question (Sup.1) to participants who answered “yes” to the above question: “What challenges do you face in providing psychological care to patients at their end of life?” Participants could respond freely to this open-ended question.

### Qualitative analyses

Content analysis was used to analyze the responses to the open ended question answered freely. Content analysis is an objective and systematic procedure used to draw conclusions by creating categories of data from verbatim or unstructured data [30]. We conducted a quantitative content analysis according to previous studies in palliative care settings [28, 31]. Our content analysis procedure was conducted as follows: (1) all text data were divided into thematic units, which are units of words with one logical meaning; (2) two researchers, a clinical psychologist (KI), and a cardiovascular nurse (SM) extracted all statements from the free descriptions related to the study topic, such as the barriers to providing end-of-life psychiatric care; (3) a clinical psychologist (KI), a cardiovascular nurse (SM), and two psychiatrists in the palliative care team (EM and TT) carefully conceptualized similarities and differences in the content, and defined all categories; and (4) two coders, a student of psychology, and a psychiatric clinical nurse independently determined how each thematic unit that was identified corresponded with any category. The concordance rate and kappa coefficient of the determinations of the categories were used as reliability indicators. The kappa coefficient was calculated using 20% of the data and random sampling was conducted based on the data from a standard set derived from a previous study, with more than 10% or 50 units of data [32, 33].

### Statistical analyses

First, we summarized the characteristics of the participants and hospitals using standard descriptive statistics. Second, the mean difference in difficulties in providing palliative care was compared between oncological and cardiovascular hospitals using a *t* test, and the frequency of difficulties in providing end-of-life psychiatric care was compared between oncological and cardiovascular hospitals using *χ*^*2*^ test. Third, the frequency of the thematic units that were categorized in the above content analysis was compared between health care providers in oncological and cardiovascular hospitals using *χ*^*2*^ test. The significance level was set at 5%. All data were analyzed using IBM SPSS Statistics for Windows, version 24 (IBM Corp., NY, USA).

## Results

### Demographic and clinical characteristics

**Fig. 1:**
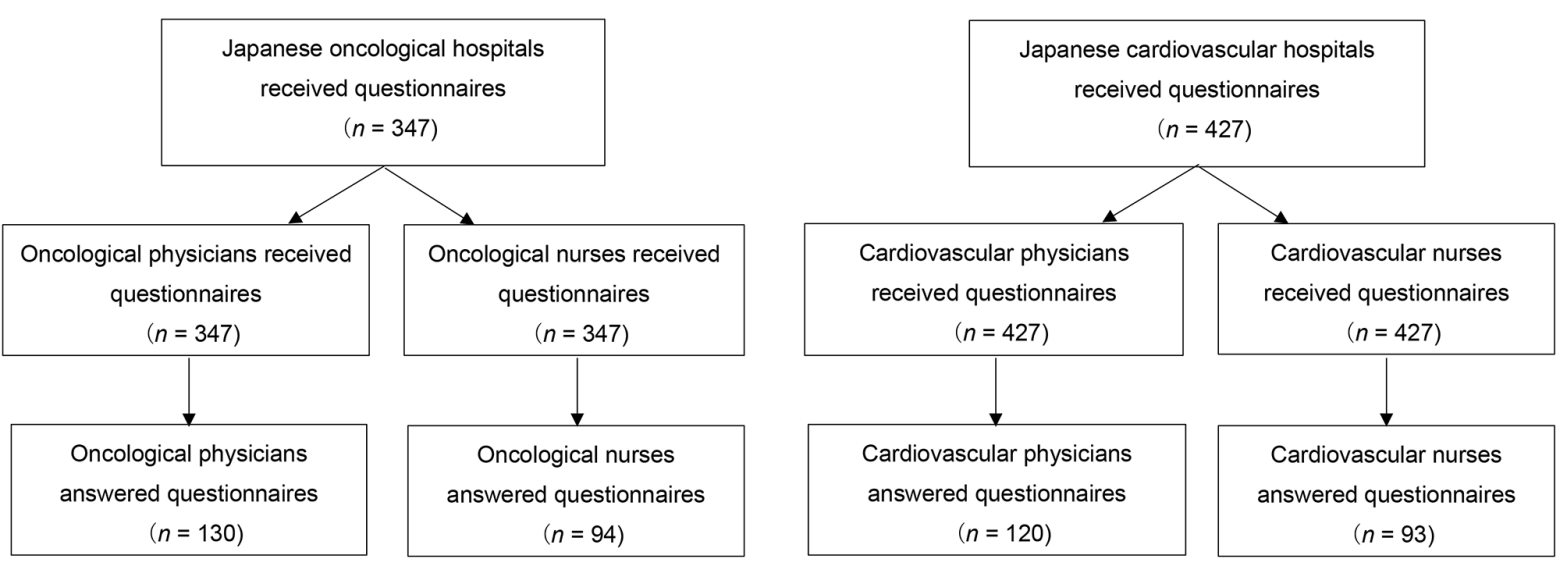
Flow diagram of participants

From the 347 oncology and 427 cardiology hospitals, 130 oncological physicians (37.5%), 94 oncological nurses (27.1%), 120 cardiovascular physicians (28.1%), and 93 cardiovascular nurses (21.8%) were included in the analysis (Fig. 1). The characteristics of the study participants and hospitals are listed in Table 1. More than 90% of physicians were specialists, such as lung cancer or cardiovascular specialists, and approximately half of the nurses were certified in a specialized field, including cancer nursing or palliative care. The sex ratio (men:women) was 1.4:1. Regarding both oncology and cardiology hospitals, more than 90% were general hospitals, approximately 60% were large-scale facilities (≥ 500 hospital beds), more than 80% had palliative care teams, and approximately 70% had psychiatric or psychological care specialists.

**Table 1:** Characteristics of participants (health care providers) and hospitals

	Oncological hospitals (*n* = 224)		Cardiovascular hospitals (*n* = 213)	
Licenses				
Physicians	130 (58.0)		120 (56.3)	
- Specialist	122 (54.5)		116 (54.5)	
Nurses	94 (42.0)		93 (43.7)	
- Certified nurse specialist	3 (1.3)		4 (1.9)	
- Certified nurse	48 (21.4)		28 (13.1)	
Sex				
Men	123 (55.9)		123 (58.6)	
Women	97 (44.1)		87 (41.4)	
Age				
21 − 30 years old	5 (2.2)		5 (2.4)	
31 − 40 years old	43 (19.3)		50 (23.7)	
41 − 50 years old	82 (36.8)		86 (40.8)	
51 − 60 years old	81 (36.3)		66 (31.3)	
>61 years old	12 (5.4)		4 (1.9)	
Area				
Hokkaido / Tohoku area	32 (15.2)		31 (15.3)	
Kanto / Koshinetsu area (except for Tokyo)	69 (32.9)		56 (27.6)	
Tokyo	17 (8.1)		16 (7.9)	
Chubu / Hokuriku area	12 (5.7)		12 (5.9)	
Kinki area	27 (12.9)		33 (16.3)	
Chugoku / Shikoku area	33 (15.7)		24 (11.8)	
Kyushu / Okinawa area	20 (9.5)		31 (15.3)	
Hospital type				
National medical center	1 (0.5)		3 (1.4)	
Academic medical center	51 (23.6)		60 (28.7)	
General hospital except academic medical center	157 (72.7)		125 (59.8)	
Specialized hospital	7 (3.2)		21 (10.0)	
Number of hospital beds				
<300	8 (3.7)		23 (10.8)	
≥300, < 500	81 (37.1)		64 (30.1)	
≥500	129 (59.2)		126 (59.2)	
Palliative care unit in hospital				
Yes	67 (30.2)		59 (27.7)	
No	155 (69.8)		150 (70.4)	
Palliative care team in hospital				
Yes	223 (100.0)		185 (87.3)	
No	0 (0.0)		22 (10.4)	
Liaison psychiatry team in hospital				
Yes	89 (40.5)		88 (41.7)	
No	114 (51.8)		106 (50.2)	
Palliative care physicians in hospital				
Yes	166 (75.5)		144 (67.6)	
No	53 (24.1)		60 (28.2)	
Psychiatrists in hospital				
Yes	177 (79.4)		165 (77.5)	
No	44 (19.7)		47 (22.1)	
Clinical psychologists in hospital				
Yes	161 (73.2)		140 (65.7)	
No	40 (18.2)		59 (27.7)	

### Difficulty in providing end-of-life palliative and psychiatric care

**Fig. 2:**
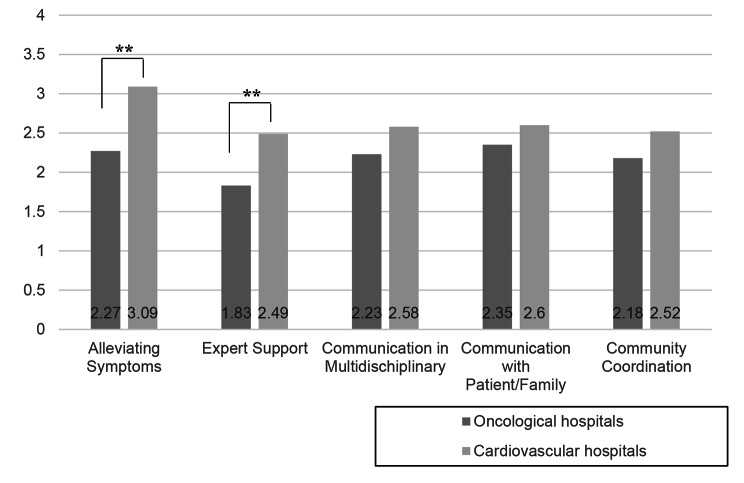
Difficulty in providing palliative care (scores)

We found that the Palliative Care Difficulties Scale scores were significantly higher in health care providers among cardiology hospitals compared to that of oncology hospitals for “alleviating symptoms” and “expert support” (*F* [423] = 8.63, *p* = 0.00 and *F* [414] = 18.96, *p* = 0.00, respectively), whereas no significant differences were found for any other factor (*F* [426] = 3.50, *p* = 0.06 for multidisciplinary communication; *F* [424] = 2.82, *p* = 0.09 for communication with patient/family; *F* [423] = 1.11, *p* = 0.29 for community coordination) (Fig. 2).

**Fig. 3:**
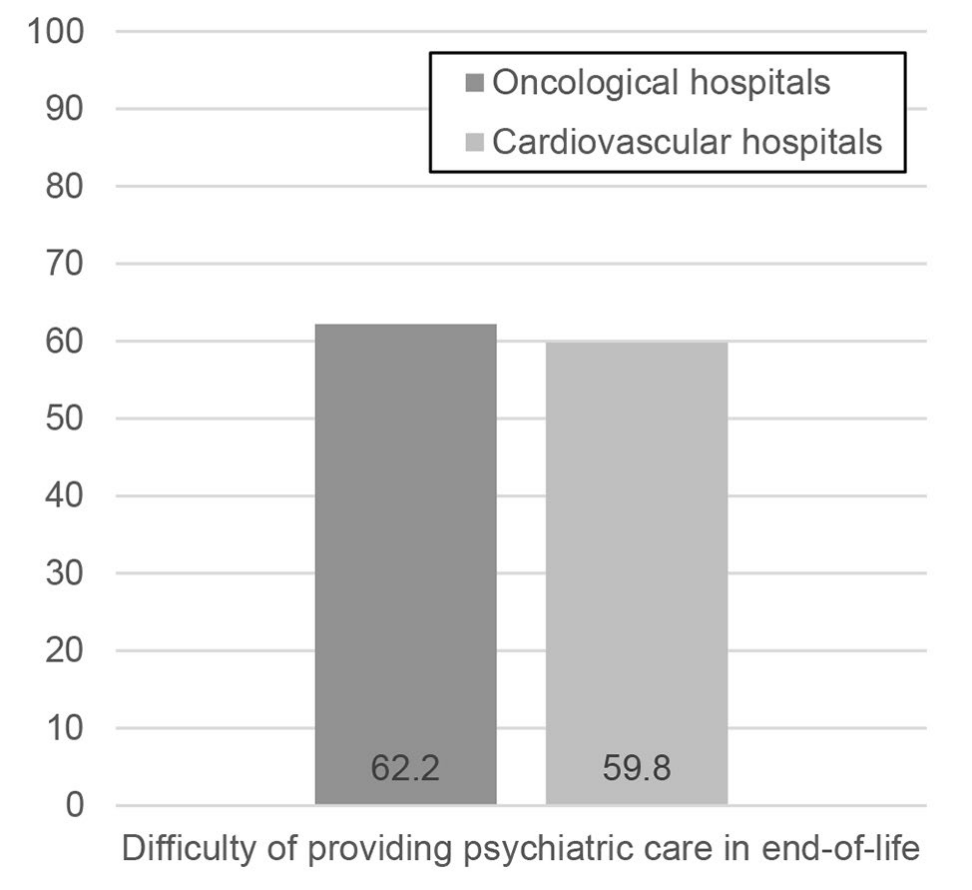
Difficulty in providing end-of-life psychiatric care (%)

The frequency of difficulties in providing end-of-life psychiatric care according to the *χ*^*2*^ test and exact probability test is shown in Fig. 3. A total of 135 (62.2%) oncological and 125 (59.8%) cardiovascular health care providers had difficulties in providing end-of-life psychiatric care. There was no significant difference in the frequency of difficulties faced by healthcare providers of oncology and cardiology hospitals (*χ*^*2*^ [1] = 0.26, *p* = 0.62).

### Barrier to providing end-of-life psychiatric care using qualitative methods

We extracted 52 attributes from the content analysis, 40 of which were classified by the semantic content into “patients’ personal problems,” “family members’ problems,” “professionals’ personal problems,” “communication problems between professionals and patients,” “problems specific to end-of-life care,” “problems specific to psychiatric care,” “problems of institution or system,” and “problems specific to non-cancer patients” (Table 2). The Kappa coefficient derived by the two independent coders was 0.54 in the random 20% data of this study.

**Table 2:** Barriers to providing end-of-life psychiatric care

	Total		Oncological hospitals (*n* = 117)		Cardiovascular hospitals (*n* = 106)	
	*n*	(%)	*n*	(%)	*n*	(%)
A: Patients’ personal problems						
1. Accepting reality and physical condition (A5)	14	(6.3)	8	(6.8)	6	(5.7)
2. Lack of emotion expression (A8)	12	(5.4)	6	(5.1)	6	(5.7)
3. Severe depression or suicide ideation (A7)	5	(2.2)	2	(1.7)	3	(2.8)
4. Problems of cognitive function or comprehension (A4)	8	(3.6)	6	(5.1)	2	(1.9)
5. Depression (A2)	8	(3.6)	4	(3.4)	4	(3.8)
6. Refusal of psychiatric care (A6)	7	(3.1)	4	(3.4)	3	(2.8)
7. Anxiety or embarrassment (A1)	5	(2.2)	5	(4.3)	0	(0.0)
8. Problems specific to young adults (A9)	4	(1.8)	3	(2.6)	1	(0.9)
9. Aggression (A3)	3	(1.3)	2	(1.7)	1	(0.9)
B: Family members’ problems						
10. Lack of family support (B2)	13	(5.8)	7	(6.0)	6	(5.7)
11. Accepting reality among family members (B1)	13	(5.8)	8	(6.8)	5	(4.7)
12. Differences in opinions or comprehension among family members (B4)	10	(4.5)	9	(7.7)	1	(0.9)
13. Problems of family relationship (B3)	3	(1.3)	1	(0.9)	2	(1.9)
C: Professionals’ personal problems						
14. Problems of time and place (C1)	33	(14.8)	18	(15.4)	15	(14.2)
15. Lack of self-confidence in one’s skills in psychiatric care (C2)	20	(9.0)	9	(7.7)	11	(10.4)
16. Problems related to inter-professional team work (C5)	14	(6.3)	8	(6.8)	6	(5.7)
17. Ability differences between health care providers (C4)	9	(4.0)	6	(5.1)	3	(2.8)
18. Psychological burden in health care providers (C3)	3	(1.3)	1	(0.9)	2	(1.9)
D: Communication problems between professionals and patients						
19. Lack of trust between professionals and patients (D2)	7	(3.1)	5	(4.3)	2	(1.9)
20. Differences in opinions or comprehension in professionals vs. patients (D1)	4	(1.8)	3	(2.6)	1	(0.9)
E: Challenges specific to end-of-life care						
21. Difficulty in providing psychological care to patients who were not disclosed the “bad news” (E5)	10	(4.5)	5	(4.3)	5	(4.7)
22. Difficulty in providing psychological care after disclosure of “bad news” (E4)	10	(4.5)	4	(3.4)	6	(5.7)
23. Circumstances do not meet patients’ expectations (E7)	6	(2.7)	1	(0.9)	5	(4.7)
24. Problems of sudden deterioration of physical condition (E2)	5	(2.2)	2	(1.7)	3	(2.8)
25. Problems of spiritual pain and acceptance of death (E3)	4	(1.8)	2	(1.7)	2	(1.9)
26. Difficulty in providing psychological care to patients who were disclosed the “bad news” at a later time (E6)	2	(0.9)	0	(0.0)	2	(1.9)
27. Problems of physical pain (E1)	2	(0.9)	1	(0.9)	1	(0.9)
F: Challenges specific to psychiatric care						
28. Necessity of individual care for each patient (F2)	14	(6.3)	7	(6.0)	7	(6.6)
29. Difficulty of psychiatric assessment and intervention (F1)	7	(3.1)	3	(2.6)	4	(3.8)
30. Lack of robust policy or correct answer (F3)	2	(0.9)	1	(0.9)	1	(0.9)
G: Problems of institution or system						
31. Lack of professional team or health care providers (G1)	12	(5.4)	4	(3.4)	8	(7.5)
32. Lack of training system for psychiatric care (G3)	7	(3.1)	1	(0.9)	6	(5.7)
33. Short-handed conditions (G2)	6	(2.7)	5	(4.3)	1	(0.9)
34. Difficulty of compatibility with outpatient service (G4)	5	(2.2)	5	(4.3)	0	(0.0)
35. Difficulty of participating in informed consent (G5)	4	(1.8)	3	(2.6)	1	(0.9)
H: Challenges specific to non-cancer patients						
36. Difficulty in evaluating prognostic prediction in non-cancer patients (H2)	10	(4.5)	0	(0.0)	10	(9.4)
37. Lack of practice guidelines for non-cancer patients (H1)	4	(1.8)	0	(0.0)	4	(3.8)
38. Lack of understanding about providing palliative care for non-cancer patients in patients or family members (H3)	3	(1.3)	0	(0.0)	3	(2.8)
39. Lack of experience of health care providers in providing palliative care to non-cancer patients (H4)	4	(1.8)	1	(0.9)	3	(2.8)
40. Lack of a professional team or health care providers who specialize in palliative care for non-cancer patients (H5)	3	(1.3)	0	(0.0)	3	(2.8)

The frequency of barriers to providing psychiatric end-of-life care is shown in Table 3. We found that the “problems specific to non-cancer patients” occurred more frequently in health care providers of cardiology than that of oncology hospitals (*χ*^*2*^ [1] = 22.475, *p* = 0.00). There was no significant difference between the frequencies of any other barrier between health care providers of oncology and cardiology hospitals.

**Table 3:** Differences in barriers to providing end-of-life psychiatric care between health care providers of oncology and cardiology hospitals

	Total		Oncology hospitals (*n* = 117)		Cardiology hospitals (*n* = 106)			
	*n*	(%)	*n*	(%)	*n*	(%)	*χ* ^*2*^	*p*
A: Patients’ personal problems	56	(25.1)	33	(28.2)	23	(21.7)	1.25	0.26
B: Family members’ problems	38	(17.0)	24	(20.5)	14	(13.2)	2.10	0.15
C: Professionals’ personal problems	65	(29.1)	30	(25.6)	35	(33.0)	1.47	0.23
D: Communication problems between professionals and patients	11	(4.9)	8	(6.8)	3	(2.8)	1.91	0.17
E: Problems specific to end-of-life care	34	(15.2)	13	(11.1)	21	(19.8)	3.26	0.07
F: Problems specific to psychiatric care	23	(10.3)	11	(9.4)	12	(11.3)	0.22	0.64
G: Problems of institution or system	32	(14.3)	17	(14.5)	15	(14.2)	0.01	0.94
H: Problems specific to non-cancer patients	22	(9.9)	1	(0.9)	21	(19.8)	22.48	0.00^*^

^*^p < 0.05

## Discussion

This is the first study that investigated the barriers to providing psychiatric care for end-stage HF patients compared to end-stage cancer patients. Although we found no significant difference in the frequency of those who perceive barriers to providing end-of-life psychiatric care between the cardiology and oncology settings, there can be a difference in the context in which they perceive barriers. A particularly important result was that the cardiovascular health care providers faced problems with psychiatric care, which were specific to non-cancer patients, such as obtaining professional support, useful guidelines, or training opportunities. This study was useful in exploring solutions for providing sufficient psychiatric care for end-stage HF patients, by eliminating barriers using a bottom-up qualitative approach.

Our results indicated that there were three challenges faced by health care providers in providing psychiatric care to end-of-life patients. First, knowledge of mental health issues specific to the end-of-life is necessary for health care providers to provide psychiatric care. Cardiovascular health care providers found it particularly difficult to improve their knowledge and skills for performing psychiatric assessments and for treating psychological and cardiac symptoms. In particular, depression, in addition to fatigue or pain, is one of the most common symptoms and imposes a heavy burden on patients with advanced HF [12, 13, 34]. Some clinical practice guidelines on HF emphasize the need for psychiatric care for HF patients with depression as part of symptom management in Western countries [5, 35]. However, even these guidelines have insufficient information about a specific psychiatric assessment and treatment for patients with HF. Participants in this study also described that they had little access to information needed to improve their knowledge and skills in psychiatric care. For cancer patients, lack of knowledge and training among health care providers is a barrier to providing psychiatric care [36], and therefore some Japanese academic societies have held seminars or workshops to promote psychiatric care knowledge for oncologists or any other health care providers in the last few decades. Taken together, we recommend an expansion of the existing training and education system and provision of detailed guidelines as a way to provide access to methods of psychiatric assessment and treatment for psychological symptoms in patients with advanced HF. Furthermore, physical symptom management was also identified as a difficulty for cardiovascular health care providers compared with oncological health care providers in this study. Interventions directed at alleviating physical symptoms related to HF can lead to a reduction in psychological symptoms in palliative care [37]. In the future, we recommend the development of a training system for end-of-life care professionals aimed at providing training for both physical and psychiatric care.

Second, cooperation among health care providers with different specialties is important in providing psychiatric care for end-stage patients. Many health care providers felt that it was difficult to coordinate professional-patient relationships in both cardiovascular and oncological settings. Interventions to enhance communication between professionals and patients can improve the latter’s psychological well-being [38]. Professional-patient relationship and communication are also important for the quality and outcome of medical treatment [39, 40]. Particularly in palliative settings, a lack of communication between professionals and patients can lead to the inhibition of critical decisions such as ICD deactivations [41, 42]. Practically, general education and specialized education can improve communication skills among health care providers and facilitate professional-patient communication [43, 44]. Advanced care planning can also encourage effective communication between professionals and patients with HF [45, 46]. Therefore, we conclude that a useful tool or training system for improving communication skills as well as psychiatric care skills among health care providers could enhance end-of-life care in cardiovascular settings.

Third, health care providers’ own difficulties and distresses can be resolved to implement psychiatric care smoothly for end-stage patients. A professional’s personal psychological or physical distress could be a barrier to providing psychiatric care. Professional participants in this study described that many cardiovascular and oncological hospitals do not have sufficient staff and are consequently overwhelmed by the workload, leading to unsatisfactory psychiatric care for palliative patients. Health care providers also feel unable to provide sufficient spiritual psychiatric care for end-of-life patients [47]. Reducing the workload and ensuring adequate time management for health care providers remain critical goals in modern Japanese medical settings.

### Limitations

Our study has three major limitations. First, recall bias may have occurred because of the self-reported nature of the questionnaires. However, we conducted a content analysis by two researchers independently and ensured objectivity. Second, although the study conducted on a nation-wide level in Japan, the data may not be generalizable to other populations of the world. Therefore, future studies investigating the same research questions in other countries will be essential to validate our findings and to add to the evidence database. Third, as this study was conducted before the COVID-19 pandemic, our findings may not be consistent with the current situation in the Japanese medical field. Although it is noteworthy that the medical field is constantly overwhelmed with achieving a level of infection control, and the perception of health care providers regarding the significance of providing psychiatric care at the end of life is also changing.

## Conclusion

Our results demonstrated that (1) both cardiovascular and oncological health care providers perceive the barriers to providing end-of-life psychiatric care; (2) both of them faced challenges in terms of patients’ personal problems, family members’ problems, professionals’ personal problems, communication problems between professionals and patients, problems specific to end-of-life care, problems specific to psychiatric care, problems of institution or system, and problems specific to non-cancer patients; and (3) cardiovascular providers particularly faced challenges specific to non-cancer patients, compared to oncology providers.

These results suggest that health care providers in cardiovascular hospitals, in contrast to those in oncological hospitals, experience problems in obtaining useful guidelines or training opportunities. We recommend the staffing to provide adequate psychiatric care for end-stage HF patients, and the provision of continuous educational opportunities for health care providers involved with psychiatric and palliative care for patients with HF. However, our study also indicates that both oncological and cardiovascular health care providers face challenges in providing end-of-life psychiatric care, which stem from patients’ or health care providers’ personal problems, among others. Therefore, we should also develop strategies to overcome not only the understaffing situation in medical services but also a lack of professionals’ psychiatric care skills.

## Electronic supplementary material

Below is the link to the electronic supplementary material.

Sup. 1 The questionnaire English translated version

## Data Availability

The datasets generated and/or analyzed during the current study are not publicly available as this permission was not obtained in the informed consent form, however, data are available from the corresponding author on reasonable request.
