# Supplementary material for: Health care providers’ perspectives on providing end-of-life psychiatric care in cardiology and oncology hospitals: a cross-sectional questionnaire survey
Source: BMC Palliat Care. 2023 Mar 15;22:23. doi: 10.1186/s12904-023-01138-z (PMC10014396; doi:10.1186/s12904-023-01138-z)
Supplement: Supplementary file 1 — Sup. 1 The questionnaire English translated version [file 12904_2023_1138_MOESM1_ESM.docx]

**Sup. 1** The questionnaire English translated version

| 1. Do following health care professionals provide psychiatric care for patients in their end of life? 2. Physicians：　□_1_ Always　　□_2_ Sometimes　　□_3_ Nothing 3. Nurses：　□_1_ Always　　□_2_ Sometimes　　□_3_ Nothing 4. Psychiatrists：　□_1_ Always　　□_2_ Sometimes　　□_3_ Nothing 5. Psychologists：　□_1_ Always　　□_2_ Sometimes　　□_3_ Nothing 6. Palliative care team：　□_1_ Always　　□_2_ Sometimes　　□_3_ Nothing 7. Liaison psychiatry team：　　□_1_ Always　　□_2_ Sometimes　　□_3_ Nothing |
| --- |
| 1. Do you face challenges in providing psychiatric care for patients at their end of life?   　　　　　□_1_ Yes　　　□_2_ No |
| 1. What challenges do you face in providing psychological care to patients at their end of life? (free-text entry) |
